# An integrative approach to identifying cancer chemoresistance-associated pathways

**DOI:** 10.1186/1755-8794-4-23

**Published:** 2011-03-24

**Authors:** Shih-Yi Chao, Jung-Hsien Chiang, A-Mei Huang, Woan-Shan Chang

**Affiliations:** 1 Department of Computer Science and Information Engineering, Ching Yun University, No. 229, Jiansing Road, Jhongli City, Taoyuan County 320, Taiwan; 2 Department of Computer Science and Information Engineering, National Cheng Kung University, No. 1, University Road, Tainan City 701, Taiwan; 3 Department of Biochemistry, Kaoshiung Medical University, Shih-Chuan 1st Road, Kaohsiung, 807, Taiwan

## Abstract

**Background:**

Resistance to chemotherapy severely limits the effectiveness of chemotherapy drugs in treating cancer. Still, the mechanisms and critical pathways that contribute to chemotherapy resistance are relatively unknown. This study elucidates the chemoresistance-associated pathways retrieved from the integrated biological interaction networks and identifies signature genes relevant for chemotherapy resistance.

**Methods:**

An integrated network was constructed by collecting multiple metabolic interactions from public databases and the k-shortest path algorithm was implemented to identify chemoresistant related pathways. The identified pathways were then scored using differential expression values from microarray data in chemosensitive and chemoresistant ovarian and lung cancers. Finally, another pathway database, Reactome, was used to evaluate the significance of genes within each filtered pathway based on topological characteristics.

**Results:**

By this method, we discovered pathways specific to chemoresistance. Many of these pathways were consistent with or supported by known involvement in chemotherapy. Experimental results also indicated that integration of pathway structure information with gene differential expression analysis can identify dissimilar modes of gene reactions between chemosensitivity and chemoresistance. Several identified pathways can increase the development of chemotherapeutic resistance and the predicted signature genes are involved in drug resistant during chemotherapy. In particular, we observed that some genes were key factors for joining two or more metabolic pathways and passing down signals, which may be potential key targets for treatment.

**Conclusions:**

This study is expected to identify targets for chemoresistant issues and highlights the interconnectivity of chemoresistant mechanisms. The experimental results not only offer insights into the mode of biological action of drug resistance but also provide information on potential key targets (new biological hypothesis) for further drug-development efforts.

## Background

The development of chemotherapy resistance is of tremendous significance to patients, researchers, and care providers who rely on conventional cytotoxic agents for the treatment of cancer. Still, the mechanisms and related biological pathways that contribute to chemotherapy resistance are relatively poorly understood. Numerous attempts have been made to mitigate or eliminate chemotherapy resistance, based-on certain assumptions about the various mechanisms, but low response rates and poor clinical outcomes for patients can be attributed to our inability to identify and subsequently target major molecular interactions associated with such resistance. Many genes have recently been reported to determine sensitivity to multiple drugs include drug transporters and metabolizing enzymes [1-4], and certain genes have also been demonstrated to determine sensitivity to specific chemotherapy drugs [5-7]. Other studies have attempted to estimate the chemosensitivity of cancers using genome-wide expression profile analyses, such as cDNA microarray and single nucleotide polymorphisms [8-10]. Although these studies have described genes as being capable of determining the sensitivity to chemotherapy drugs, the interactions between such genes have not been addressed, and considerable attention has focused on identifying molecular interactions associated with chemotherapy resistance. Cabusora *et al*. reported particular response sub-networks in the *M. tuberculosis *network after treatment with unspecific stress-inducers and comparison with antibacterial drugs [11]. To identify rational targets for combination therapy, Riedel *et al*. attempted to identify the biological networks implicated by differential gene expression between sensitive and resistant cell lines [12].

However these studies did not take into account the drug active pathways, including the regulatory interactivities of genes influenced by the drug. The drug active pathway plays an important role in the drug responses of the cellular system affected by the drug and the prediction of side-effects, which is also a very important issue for identifying and validating drug target genes through their regulatory relationships. Moreover, considerations should be taken of drug resistance mechanisms, including reduced intracellular drug accumulation, increased detoxification of the drug by thiol-containing molecules, increased DNA damage repair, and altered cell signaling pathways and apoptosis mediators [13]. In addition, chemotherapy drugs can be categorized based on their function, chemical structure and interaction with other drugs. Cisplatin and carboplatin, classified as DNA alkylating agents, are platinum-based chemotherapy drugs used to treat various cancers, including sarcomas, small cell lung cancer, ovarian cancer, lymphomas and germ cell tumors. These platinum-based chemotherapy drugs react with DNA *in vivo *by binding to and causing cross-linking of DNA which ultimately triggers apoptosis [14]. For example, cisplatin forms highly reactive, charged, platinum complexes which bind to nucleophilic groups (such as GC-rich sites) in DNA, inducing intra-strand and inter-strand DNA cross-links, as well as DNA-protein cross-links. These cross-links result in apoptosis and cell growth inhibition. When cells become resistant to cisplatin, the doses must be increased, and a large dose escalation can lead to severe multi-organ toxicities and intractable vomiting. The mechanisms of cisplatin drug resistance may include decreased intracellular accumulation of cisplatin and increased DNA repair, which also are drug resistance related pathways considered in this approach. Hence, a large biological interaction network was re-constructed by collecting from public databases DNA damage-related pathways, cell signalling-related pathways and the regulatory relationships between genes.

Combining pathway structure information mined from the re-constructed large biological interaction network with gene differential expression values, this study elucidates the particular platinum-based chemoresistance-associated pathways. Genes deemed relevant for chemotherapy resistance were also determined. Results of this study demonstrated that the identified pathways can increase chemotherapy resistance. This approach can identify pathways with a response dissimilar to that of known modes of biological action, and these new hypotheses can be used early in the drug development process to avert repeated and costly clinical trails. The major contributions of this approach are: (1) to reveal the phenomenon of chemoresistant mechanisms and related interactions between genes by combining pathway structure information with gene differential expressions; (2) to provide crossing validation candidate signature gene sets by calculating the values of betweenness centrality and degree in large complex networks; and (3) to propose new hypotheses for chemoresistant mechanisms through systems biology.

## Methods

### Materials and databases

This section covers the graph-theoretical properties, biological network constructions, and data sets.

#### Graphs and networks

Basic graph-theoretical properties and representations used by this study are as follow:

**DEFINITION**. A graph *G *= (*V*, *E*) = (*V*(*G*), *E*(*G*)) consists of a vertex set *V*(*G*) with vertices (or nodes) *v_i _*∈ *V*(*G*), and an edge set *E*(*G*) with (*v_i_*, *v_j_*)∈*E*(*G*).

A graph *G *with biological information yields a biological network *N_B _*as follows:

**DEFINITION**. Let *N_B _*= (*V*, *E*, *δ*) be a network with vertices *v*∈*V*, edges *e*∈*E*, and a function *δ*: *Y *→ *P *(*Y *= *V *∪ *E*) that maps vertices and edges onto their respective properties *p*∈*P*.

Depending on the particular network representation, in a biological network vertex properties can include genes, proteins or chemical elements, and edge properties may refer to specific interactions, such as binding or regulating. The mapping *δ*: *Y *→ *P *is at least subjective because for all *p*∈*P*, there exists a *y*∈*Y *with *δ*(*y*) = *p*.

#### Heterogeneous biological network integration and re-construction

To integrate heterogeneous biological networks, we identified three types of interactions relevant to a network: (i) protein interactions, such as protein-DNA binding or multi-state protein phosphorylation by kinases during signaling, (ii) regulatory reactions including co-expressions in regulons, and positive and negative regulation, and (iii) metabolic reactions. For protein interaction data, we parsed the Pathway Interaction Database (PID) [15], a highly-structured, curated collection of information about known biomolecular interactions and key cellular processes assembled into signaling pathways. Furthermore, the TRANSFAC [16] database provided information on regulatory reactions including co-expressions in regulons, and positive and negative regulation. For metabolic reaction data, we used the Kyoto Encyclopedia of Genes and Genomes (KEGG) [17,18] to construct molecular interaction and reaction networks for metabolism. KEGG contains reaction networks of cellular processes, human diseases and drug development. Given this study's focus on identifying differential expression pathways during platinum-based chemotherapy drugs resistance, we determined diversified pathways correlated with cancer diseases, DNA repair, and metabolism for parsing and integration. Pathway selection criteria and the overall pathway sets collected in this study are listed in Additional file 1.

**Figure 1:**
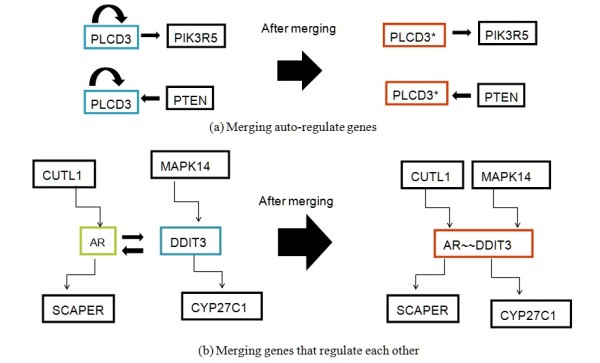
**An overview of merging vertices**. Figure 1(a) shows the concept of merging auto-regulate genes. From a biological viewpoint of transcriptional relationships, a number of genes may regulate themselves or regulate each other, resulting in cyclic relationships while re-constructing the large network, which makes it more difficult to determine simple shortest paths. Figure 1(b) demonstrates how to merge genes that regulate each other. Transcription factors, AR (androgen receptor) and DDIT3 (DNA-damage-inducible transcript 3), regulate their target genes and regulate each other as well. To preserve the biological truth and avoid loops being represented in the graph, vertices AR and DDIT3 were merged during the shortest paths algorithm. Next, while scoring the identified pathways according to gene expression data, each vertex (or gene) was considered separately and identically.

Our goal was to use protein interactions and regulatory reactions assembled into metabolic pathways without introducing duplicated links and elements. To merge interactions from various sources, the genes' alias names must be arranged in advance. Furthermore, we recorded the directions of interactions between genes (or proteins) as well to the graph. We joined the proteins as vertices to the integrated large network and connected them to any co-regulated genes by adding new edges. From a biological viewpoint of transcriptional relationships, a number of genes may regulate themselves or regulate each other, resulting in cyclic relationships while re-constructing the large network, which makes it more difficult to determine simple shortest paths. We dealt with this problem by merging vertices as demonstrated in Figure 1. Taking Figure 1(b) as an example, the transcription factors AR (androgen receptor) and DDIT3 (DNA-damage-inducible transcript 3) regulate their target genes and regulate each other as well. To preserve the biological truth and avoid loops being represented in the graph, vertices AR and DDIT3 were merged during the shortest paths algorithm. Next, while scoring the identified pathways according to gene expression data, each vertex (or gene) was considered separately and identically.

#### Microarray data

Peters *et al*. presented the results of a preliminary investigation into the molecular phenotype of patient-derived ovarian tumor cells in the context of sensitivity or resistance to carboplatin [19]. They correlated chemoresponse data with gene expression patterns at the level of transcription. Primary cultures of cells derived from ovarian carcinomas of individual patients (n = 6) were characterized using the ChemoFx assay and classified as either carboplatin sensitive (n = 3) or resistant (n = 3). Three representative cultures of cells from each individual tumor were then subjected to Affymetrix gene chip analysis (n = 18) using U95A human gene chip arrays. They identified numbers of differentially expressed genes that define transcriptional differences between chemosensitive and chemoresistant cells and temporal responses to carboplatin expressed in an *ex vivo *setting. Gabriela *et al*. investigated the response to cisplatin of a panel of NSCLC cell lines and found an inverse correlation between sensitivity and damage formation resulting from this agent [20]. Further analysis of multiple alternate cellular end-points including cell cycle analysis, apoptosis and gene expression changes, revealed cisplatin damage tolerance to be a mechanism of chemoresistance in this model system. Both gene expression data sets were available through the Gene Expression Omnibus (GEO) at NCBI [21] (GEO platform accession number GDS 1381 and GSE 6410, respectively).

### Systems and implementation

#### System overview

**Figure 2:**
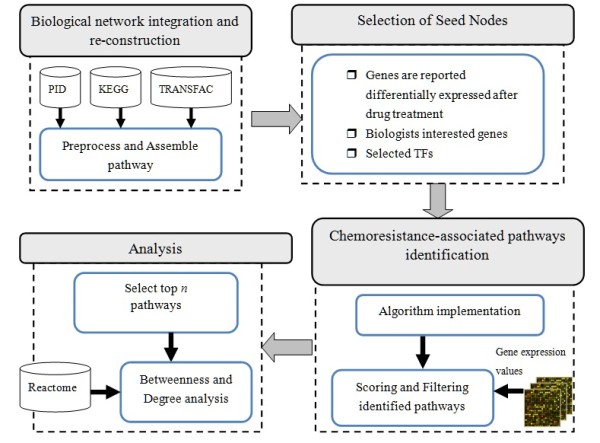
**System architecture**. The system is composed of four major parts, including heterogeneous biological network integration, seed nodes selection, pathways identification, and differential expression analysis. The large integrated biological network was constructed and stored in MySQL database. By stripping away unambiguous vertices according to the genes' official symbols and the duplicated interactions between them, the k-shortest path algorithm could be implemented to obtain the shortest pathways for given seed nodes. The seed nodes are particular nodes given by users or selected from transcription factors, and paths between them are identified by the k-shortest path algorithm. The identified pathways were scored using gene expression values as metrics for weighted edges. Finally, the top scoring n pathways were selected and further analyzed.

A system flow diagram of the corresponding processes is shown in Figure 2. The system is composed of four major parts, including heterogeneous biological network integration, the selection of seed nodes, identification of pathways, and analysis of differential expressions. As described in the previous section, the large integrated biological network was constructed and stored in MySQL database. By stripping away unambiguous vertices according to the genes' official symbols and the duplicated interactions between them, the k-shortest path algorithm could be implemented to obtain the shortest pathways for given seed nodes. The seed nodes are particular nodes given by users or selected from transcription factors, and paths between them are identified by the k-shortest path algorithm. The identified pathways were scored using gene expression values as metrics for weighted edges. Finally, the top scoring n pathways were selected and further analyzed.

#### Pathway identification

The first step in the pathway identification process was seed node selection. Here, particular vertices were tagged as "seed nodes", and the shortest paths between them were identified by Yen's algorithm [22]. From a mathematical viewpoint, this procedure extracts from the large, integrated biological network a pathway that is spanned by selected seed nodes. Yen's algorithm is still the best known approach to the k-shortest simple paths problem with respect to its worst case running time, i.e., O(*kn*(*m *+ *n*log *n*)) time for a graph with m vertices and n edges. Seed nodes were determined either by users' interesting genes [23] or were selected by biologists in advance. The criteria of selecting seed nodes are listed as follows: (i) genes with functional annotations such as 'DNA damage', 'DNA repair' and other related functional annotations [24], (ii) genes that are known transcription factors and are implicated in drug resistance [25], and (iii) genes that have been reported to have significantly altered expression patterns between platinum-based drugs chemosensitive and chemoresistant cells. We were specifically interested in a transcription factor CEBPD (CCAAT/enhancer binding protein (C/EBP), delta) which has been implicated in tumor suppression [26-29]. Interestingly, CEBPD exhibits a pro-oncogenic function in cisplatin resistance phenotype [23]. Therefore, we conducted a gene expression study to further identify CEPBD-regulated genes which might contribute to cisplatin resistance (Huang, *el al*. unpublished data). The merged CEBPD-regulated genes are listed as user-interested genes in Table 1.

**Table 1:** Lists of seed nodes

User interested gene symbols	⟡ CEBPD (CCAAT/enhancer binding protein (C/EBP), delta) ⟡ SOD1(superoxide dismutase 1, soluble) ⟡ XRCC4 (X-ray repair complementing defective repair in Chinese hamster cells 4) ⟡ PTGS2 (prostaglandin-endoperoxide synthase 2 (prostaglandin G/H synthase and cyclooxygenase), (COX2)) ⟡ RBMS3 (RNA binding motif, single stranded interacting protein) ⟡ STK39 (serine threonine kinase 39 (STE20/SPS1 homolog, yeast)) ⟡ CUTL1 (cut-like homeobox 1) ⟡ CREG1 (cellular repressor of E1A-stimulated genes 1) ⟡ APBB2 (amyloid beta (A4) precursor protein-binding, family B, member 2) ⟡ ADAMTS1(ADAM metallopeptidase with thrombospondin type 1 motif, 1) ⟡ JAZF1(JAZF zinc finger 1) ⟡ JMJD2C (Jumonji domain 2) ⟡ MSI2 (musashi homolog 2 (Drosophila)) ⟡ RABGAP1L (RAB GTPase activating protein 1-like) ⟡ NAV2 (neuron navigator 2) ⟡ ZMIZ1 (zinc finger, MIZ-type containing 1) ⟡ ZNF291 (SCAPER, S-phase cyclin A-associated protein in the ER) ⟡ ZRANB3 (zinc finger, RAN-binding domain containing 3) ⟡ CENTG2 (AGAP1, Homo sapiens ArfGAP with GTPase domain, ankyrin repeat and PH) ⟡ ATXN1 (ataxin 1-like) ⟡ THSD4 (thrombospondin, type I, domain containing 4) ⟡ CYP27C1 (cytochrome P450, family 27, subfamily C, polypeptide 1)
**Resistance genes**	⟡ IL1A (interleukin 1, alpha) ⟡ IL1B (interleukin 1, beta) ⟡ NFKB1 (nuclear factor of kappa light polypeptide gene enhancer in B-cells 1) ⟡ NFKB2 (nuclear factor of kappa light polypeptide gene enhancer in B-cells 2) ⟡ CDK4 (cyclin-dependent kinase 4) ⟡ MCM2 (minichromosome maintenance complex component 2) ⟡ MCM4 (minichromosome maintenance complex component 4) ⟡ CDC45L (CDC45 cell division cycle 45-like (S. cerevisiae))

**DNA damage genes**	⟡ MYC (v-myc myelocytomatosis viral oncogene homolog (avian)) ⟡ TP53 (tumor protein p53) ⟡ PCNA (proliferating cell nuclear antigen) ⟡ TP73 (tumor protein p73) ⟡ ATF4 (activating transcription factor 4 (tax-responsive enhancer element B67))

Seed nodes were determined either by users' interesting genes [23] or were selected by biologists in advance. The criteria of selecting seed nodes are listed as follows: (i) genes with functional annotations such as 'DNA damage', 'DNA repair' [24], (ii) genes that are known transcription factors and are implicated in drug resistance [25], and (iii) genes that have been reported to have significantly altered expression patterns between platinum-based drugs chemosensitive and chemoresistant cells.

#### Scoring and filtering pathways

The main procedure of pathway scoring was calculating the differential expression values for the genes as metrics for weighted edges in the pathway. In this study, genes, proteins and other cellular components were coded as vertices which are connected by their edges to represent the interactions in the integrated biological network. However, the scoring step assumes weights on the edges for summing scores, and such edge weights must be calculated from the vertices' scores. Therefore, the identified pathway was subsequently transformed and represented as a line graph in which the edges represent genes, proteins and other cellular components, and vertices refer to interactions. Edges can then be directly weighted by gene expression values.

**REMARK**. Give a biological network *N_B _*, its line graph *L*(*N_B_*) is a graph such that each vertex of *L*(*N_B_*) represents an edge of *N_B_*; and two vertices of *L*(*N_B_*) are adjacent if and only if their corresponding edges share a common endpoint in *N_B_*.

To filter and identify the "significant pathways" (e.g., connected sets of genes with high levels of differential expression) we followed Ideker *et al*.'s statistical scoring system which captures the amount of gene expression change in a given pathway [30]. To rate the biological activity in a particular pathway, we first assessed the significance of the differential expression for each gene. We extracted the *p*-value *p_m _*for each expressed gene *m *in the microarray data and then converted the *p_m _*into a *z*-score by Formula 1.(1)

where Φ^-1 ^denotes the inverse normal cumulative distribution function. In random data, *p*-values are distributed uniformly from 0 to 1 and *z*-scores follow a standard normal distribution, with smaller *p*-values corresponding to larger *z*-scores. The aggregate score of a set of genes in a pathway can be calculated by summing the *z_m _*over all *m *in the pathway,(2)

Under this scoring function, the pathways of all sizes can be compared, with a high score indicating a biologically active pathway and pathways were then filtered by an assigned threshold score. In summary, the *k*-shortest path approach guarantees effective pathway identification through a particular set of seed nodes. The scoring functions (Formulas 1 and 2) contribute an appropriate constraint filtering pathways. Once the top *n *pathways have been selected, the analysis of pathways process can be performed.

#### Analyze the pathway signatures

The main purpose of performing pathway intersections is to determine whether different cancers have identical chemoresistant mechanisms. Comparing two pathways requires the identification of the corresponding vertices. The correspondences between vertices in the pathways are given by matching the genes' official symbols. In general, the correspondences can be many-to-many for the reason that a vertex (e.g. representation of an enzyme) may catalyze different reactions in the pathway and may be catalyzed by multiple vertices as well. In other words, graph comparison is an NP-hard problem which typically can only be addressed by exhaustive enumeration techniques. Here, we present an approach using the vertices and edges labels of the given pathways. Consider two graphs, *N*^1^_B _(*V*_1_, *E*_1_) and *N*^2^_B _(*V*_2_, *E*_2_), and a matrix representing the correspondences between *V*_1 _and *V*_2_. Let *s*(*e*) and *t*(*e*) be the origin and terminal nodes of edge *e *so the intersection of the pathways is defined as:(3)

where *v_1_*∈*V*_1_, *v*_2_∈*V*_2 _and *e*_1_∈*E*_1_, *e*_2_∈*E*_2_. In other words, the intersection between all *v_1_*∈*V*_1 _and *v*_2_∈*V*_2_, and the intersection between corresponding edges *e*_1_∈*E*_1_, *e*_2_∈*E*_2 _under the condition that ∀ *v_1_*, *v_2_*: δ(*v_1_*) = δ(*v_2_*) and ∀ *e_1_*, *e_2_*: δ(*e_1_*) = δ(*e_2_*). An edge *e*∈ *N*^1^_B _is selected if both the originating and terminating vertices have δ-corresponding vertices in *N*^2^_B_.

To assess the importance of genes within each filtered pathway, we also implemented the betweenness centrality and degree centrality for each node. The degree and betweenness centrality of genes were calculated using the Reactome database [31] as a base to cross validate our experimental results. The betweenness centrality of a node in a network topology measures how many shortest paths go through that node. If *b_i _*is the ratio of the number of shortest paths between a pair of nodes in the network that pass through node *i *and the total number of shortest paths between those two nodes, the unscaled betweenness of node *i *is , and the (scaled) betweenness centrality is(4)

where *n *is the number of nodes in the network. The betweenness centrality is positive and always less than or equal to 1 for any network. The degree of a node in a network is the number of connections or edges by which the node is related with other nodes. Degree centrality is the number of links that connect the node to the network divided by the number of nodes in the network minus 1. It is a local measure that does not account for network context. However, changes in nodes with high degree centrality are likely to influence a large number of nodes in the network. The degree centrality was calculated by Formula (5).(5)

Formula (5) indicates the degree centrality of an undirected graph. As for a vertex representing the gene (or protein) in an undirected graph, the higher the degree, the more reactions it interacts with and the more important the vertex is.

## Results and Discussion

As described in previous section, we integrated the PID, KEGG and TRANSFAC public databases, and further eliminated duplicated reactions and elements. Accordingly, 8173 genes and 9308 interactions were retained, for which both detailed and summarized database results are presented in Table 2. In the next section, we present the experimental results and analysis of the pathway intersections.

**Table 2:** Lists of # of genes and relations in integrated database

*Statistics information of integrated databases*			
**Database**	**Organism**	**# of gene**	**# of relation**

**PID+KEGG+TRANSFAC**	Homo sapiens	8173	9308

**Reactome**	Homo sapiens	538	31240

***Statistics information on each of the three databases***			

**Database**	**# of TFs**	**# of target gene parsed**	**# of pairing regulate relation parsed**

TRANSFAC	157	825	529625

**Database**	**# of pathways**	**# of gene, protein, enzyme parsed**	**# of relation parsed**

PID + KEGG	197	18937	8880

PID	60		

KEGG	137		

We integrated the PID (the date of version, July 15, 2008), KEGG (release 47.0, July 1, 2008) and TRANSFAC public databases (version 7.0), and further eliminated duplicated reactions and elements. Accordingly, 8173 genes and 9308 interactions were remained. To assess the importance of genes within each filtered pathway, we also implemented the betweenness centrality and degree centrality for each node. The degree and betweenness centrality of genes were calculated using the Reactome database [31] as a base to cross validate our experimental results. Pathways downloaded from PID and KEGG were parsed by batch processing. A gene (or protein) may be involved in several pathways, which means some genes were repeated. Therefore, the number of parsed entity (including genes, proteins, and enzymes) was 18937. Moreover, one gene may be regulated by several TFs, or one TF may regulate numerous target genes. As a result, the total number of pairing regulate relation parsed was 529625.

### Significant pathways in ovarian cancer

**Figure 3:**
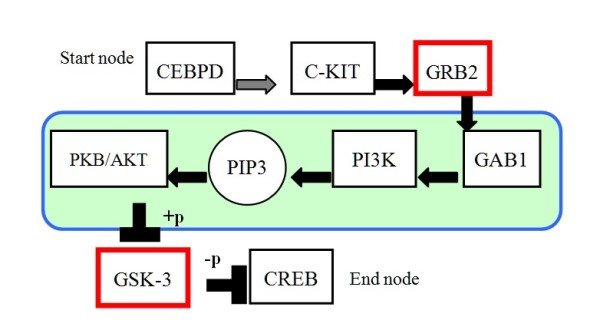
**One experimental result of identified pathways from ovarian expression data**. This diagram shows one of identified signature pathways. Genes represented by red squares indicate the connected nodes; that is, these genes connect pathways. Connected nodes are key factors for joining two or more metabolic pathways or passing down signals. The blue square indicates the PI3K/AKT pathway. The PI3K/AKT cascade plays an important role in the resistance of ovarian cancer cells to cisplatin *in vitro*. This pathway is therefore an attractive target for the development of novel anticancer agents.

Ovarian cancer is among the most malignant of all lethal diseases in women. Currently, the preferred treatment regimen for ovarian cancer is combination chemotherapy primarily with platinum-based drug such as cisplatin or carboplatin. While this treatment course has shown promising effects in a high percentage of cases, the development of chemoresistance is a significant hurdle to successful treatment outcomes [32]. Hence, we have focused our research on elucidating the mechanisms induced by chemotherapeutic agents; that is, the DNA damage, DNA repair, and apoptosis in ovarian cancer cells resulting from platinum-based drug chemotherapy and chemoresistance. One of the significant pathways identified from the ovarian cancer expression data is shown in Figure 3, with the notations presented in Additional file 2.

As shown in Figure 3, c-KIT (also called KIT or C-kit receptor) is one of target genes regulated by CEBPD, a growth factor receptor exhibiting tyrosine kinase activity. Moreover, c-KIT is not only a biochemical marker; its involvement in autocrine, paracrine or endocrine growth loops may represent a molecular mechanism behind aggressive tumor growth [33,34]. Raspollini *et al*. performed an immunohistochemistry analysis of 56 patients with advanced serous ovarian carcinomas using archival paraffin-embedded specimens and demonstrated that c-KIT was expressed in ovarian carcinoma and was statistically correlated with chemotherapy resistance [35]. C-KIT expression has been shown to be statistically correlated with the progression of disease after first-line chemotherapy. Moreover, c-KIT was identified by our pathway mining procedure with p-value < 0.05 (listed in Table 3) by t-test calculated from the ovarian expression data, indicating this approach identify genes involved in chemoresistant mechanisms.

**Table 3:** Genes identified in figure 3 with p-value < 0.05 by t-test

Gene Symbol	Ovarian *p*-value	Betweenness (mean = 3.8E-4)	Degree (mean = 9.71E-4)	Connected nodes
c-KIT (v-kit Hardy-Zuckerman 4 feline sarcoma viral oncogene homolog, also called KIT or C-kit receptor)	3.53E-07	0.00178	0.006483	

GRB2 (growth factor receptor-bound protein 2)	5.74E-06	0.020155	0.023064	V

AKT2 (v-akt murine thymoma viral oncogene homolog 2)	0.022784	3.89E-04	0.002369	V

PIK3CG (phosphoinositide-3-kinase, catalytic, gamma polypeptide)	1.29E-04	9.77E-05	0.001247	

Genes listed in this table are significant in ovarian expression data with *p*-value < 0.05. Moreover, the betweenness and degree centrality of each gene are also listed. Both GRB2 and AKT2 gene are connected nodes; which indicates they pass down signals between pathways. GRB2 with significant betweenness and degree centrality indicates it has potential to act as a "hub node" in biological interaction networks and to involve in chemoresistant mechanisms as well.

As indicated in Figure 3, the PI3K (Phosphatidylinositol 3-kinas)/AKT gene family are involved as well. The PI3K pathway is stimulated as a physiological consequence of many growth factors and regulators. In addition, the activation of the PI3K pathway results in disturbances of cell growth and survival control, which contributes to a competitive growth advantage, metastatic competence and, frequently, therapy resistance [36]. Therefore, this pathway is an attractive target for the development of novel anticancer agents. The PI3K/Akt cascade plays an important role in the resistance of ovarian cancer cells to cisplatin *in vitro*. Ohta *et al*. investigated whether the inhibition of PI3K increased the efficacy of cisplatin in an *in vivo *ovarian cancer model [37]. Blocking the PI3K/Akt cascade with a PI3K inhibitor (wortmannin) increased the efficacy of cisplatin-induced inhibition of intra-abdominal dissemination and production of ascites in athymic nude mice inoculated ip with the Caov-3 human ovarian cancer cell line. In addition, wortmannin increased the efficacy of cisplatin-induced apoptosis in tumors cells. Ohta *et al*. also confirmed that wortmannin blocked Akt phosphorylation and the downstream targets of the PI3K/Akt cascade, such as BAD (Bcl-2-associated death protein) and nuclear factor-_k_B *in vivo *by immunohistochemical staining and Western blotting. Moreover, Lee *et al*. used human ovarian cancer cell OVCAR-3 and cisplatin-resistant subclone OVCAR-3/CDDP cells to study the roles of PIK3CA (alias name PI3K) and PTEN on the resistance of human ovarian cancer cells to cisplatin-induced apoptosis [38]. They systematically examined the expressions of apoptosis regulating proteins and PI3K/Akt signaling proteins, finding that OVCAR-3/CDDP cells were 4.8-fold more resistant to cisplatin than OVCAR-3 cells following 72 h exposure to the drug. This resistance correlated with reduced susceptibility to cisplatin-induced apoptosis. Apoptotic proteins were differentially expressed in the OVCAR-3/CDDP cells, resulting in the inhibition of Bax translocalization. Their experimental results indicate that the development of resistance in OVCAR-3 cells is derived from increasing PIK3CA transcription and reducing of PTEN expression. These alterations confer resistance to cisplatin through the activation of PI3K. These *in vivo *results support the proposition that our algorithm can identify chemoresistance-associated pathways.

In Figure 3, genes are represented by red squares indicating the connected nodes; that is, these genes connect two pathways. Connected nodes are key factors for joining two or more metabolic pathways or passing down signals. Taking GRB2 (Growth factor receptor-bound protein 2) as an example, L'Esperance *et al. *[39] found that upregulated genes in post chemotherapy ovarian tumors included a substantial number of genes with previously implicated in mechanisms of chemoresistance including COX2 and tumorigenesis, GRB2. As seen in Figure 3, AKT was also identified as a connected gene, and had significant betweenness centrality and degree values (shown in Table 3), indicating that AKT has potential to act as a "hub node" in biological interaction networks and be involved in chemoresistant mechanisms as well [40].

### Significant results following pathway intersections

The main analysis of this experiment focused on whether different cancers identical chemoresistant mechanisms and whether these chemoresistant mechanisms share some genes in common. After performing intersection by Formula (3), 88 pathways remained (the Additional file 3). The following sections include further analysis.

The major goals of this analysis were: (1) to explore pathways or genes involved in chemoresistant mechanisms; (2) to delineate how these genes or pathways interact with each other; (3) to test whether the *p*-values of the genes in this pathway are significantly differentially expressed; (4) to analyze the betweenness centrality (Formula 4) and degree (Formula 5) values of genes in this pathway; and (5) to identify the chemoresistance-associated genes.

**Figure 4:**
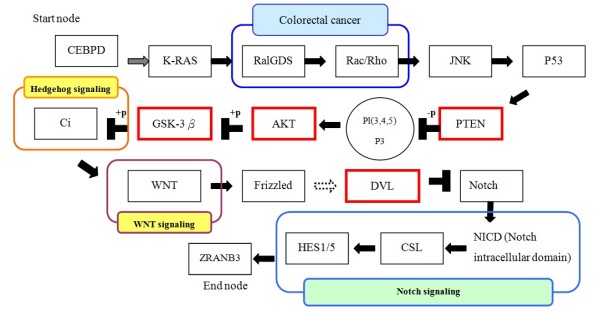
**One of experimental result from pathways intersection**. This figure demonstrates the experimental result after performing pathway interaction. Several pathways contributed to this result: the colorectal cancer related pathway, the hedgehog signaling pathway, the WNT signaling pathway and the notch signaling pathway. The connected gene DVL (disheveled, dsh homolog) connects two critical pathways: the WNT signaling pathway and the Notch signaling pathway. Gatcliffe *et al*. suggested that WNT signaling plays a role in ovarian tumorigenesis [45]. WNT signaling has a significant influence on the embryonic development of the ovary and is also involved in normal follicular development and ovarian function [46,47]. The WNT signaling pathway is involved in ovarian cancer development via multiple, diverse mechanisms, including gene mutations and changes in pathway components such as extracellular inhibitors and intranuclear transcription cofactors. According to Wang *et al*., the WNT signaling pathway passes signals to the Notch signaling pathway [48]. The Notch signaling pathway is known to be responsible for maintaining a balance between cell proliferation and death and, as such, plays an important role in the formation of many types of human tumors. In our computational results, WNT signaling connects the Notch signaling pathway through DVL gene, which indicates DVL is a critical gene for passing signals through pathways. In addition, the computational evidence provided by the values of betweenness centrality, degree and *p*-value indicate that DVL may be involved in platinum-based chemoresistance.

As shown in the Diagram 4, several pathways contributed to this result: the colorectal cancer related pathway, the hedgehog signaling pathway, the WNT signaling pathway and the notch signaling pathway. In addition, some other pathways, such as the p53 signaling pathway, the MAPK signaling pathway, and the focal adhesion were partially involved as well. Platinum-based cancer drugs (including cisplatin and carboplatin) are among the most potent anti-tumor agents, displaying clinical activity against a wide variety of solid tumors. Its cytotoxic mode of action is mediated by its interaction with DNA to form DNA adducts, primarily intrastrand crosslink adducts, which activate several signal transduction pathways, including those involving ATR, p53, p73, and MAPK, and culminate in the activation of apoptosis [41]. Resistance mechanisms that limit the extent of DNA damage include reduced drug uptake, increased drug inactivation, and increased DNA adduct repair. Mechanisms that inhibit the propagation of the DNA damage signal to the apoptotic machinery include loss of damage recognition, overexpression of HER-2/neu, activation of Akt (indicated by the red square in Figure 4), and loss of p53 function [5]. The molecular signature defining the resistant phenotype varies between tumors, and the number of resistance mechanisms activated in response to selection pressures dictates the overall extent of resistance. This experimental result implies the complicated nature of chemoresistance progression, which reflects that several mechanisms contribute to the multi-factorial nature of the chemoresistance problem. Although ovarian and lung cancers are assorted malignancies, based on the results of the pathway intersections experiment, several mechanisms are together responsible for platinum-based chemoresistance.

Table 4 shows the genes that involved in intersected pathways with *p*-value < 0.05 calculated in the expression data for ovarian cancer and lung cancer. For example, the expression values for the AKT gene, are not only significantly different in both cancer expression data sets, but the value of betweenness centrality and degree are higher than 3.8E-4 and 9.71E-4 (the respective average values). In biological terms, the betweenness centrality of a gene measures how many pathways or signal transductions go through that gene. Our experimental result indicates that the AKT gene plays an important role in chemoresistance-associated pathways. Gagnon *et al*. suggested that some Akt isoforms, such as Akt2 and Akt3, are involved in chemoresistance to cisplatin and that these isoforms could be putative targets for gene therapy for uterine cancers [42]. They performed biological experiments to demonstrate that Akt activity was directly involved in chemoresistance to cisplatin and to find Akt phosphorylation in KLE cells since it was a wild-type expressing PTEN cancer cell line. As shown in Table 4, PTEN was the first tumor suppressor gene to be identified in the phosphatase family, and the principal function of its gene product appears to be dephosphorylation of the second messenger PIP3 [43]. The expression of PTEN in two independent glioblastoma cell lines results in the disruption of downstream signaling of PI3K to Akt and Bad [44]. Thus, when PTEN is present, Akt phosphorylation is blocked and apoptosis mechanisms may be activated. The importance of Akt and PTEN genes are as well revealed by this work, which illustrates the accuracy and efficiency of our algorithm.

**Table 4:** Genes identified in figure 4 with *p*-value < 0.05 by t-test

Gene Symbol	ovarian *p*-value	lung *p*-value	Betweenness (mean = 3.8E-4)	Degree (mean = 9.71E-4)	Connected nodes
KRAS (v-Ki-ras2 Kirsten rat sarcoma viral oncogene homolog)	6.23E-04	0.001393	0.00703	0.009226	

TP53 (tumor protein p53)	0.011083	1.82E-04	0.046039	0.029049	

AKT (v-akt murine thymoma viral oncogene homolog)	1.87E-05	4.60E-06	0.009775	0.013091	V

GSK3β (glycogen synthase kinase 3 beta)	3.09E-06	1.81E-04	0.003932	0.006483	V

WNT (wingless-type MMTV integration site family)	0.009519	0.002234	1.51E-04	4.99E-04	

PTEN (phosphatase and tensin homolog)	0.001494	0.016189	0.002282	0.002618	V

DVL (dishevelled, dsh homolog 1 (Drosophila))	1.95E-08	1.80E-05	0.001653	0.002618	V

HES1 (hairy and enhancer of split 1, (Drosophila))	0.005831	2.82E-07	4.08E-04	0.001745	

Genes listed in table 4 are significant in both ovarian and lung expression data with p-value < 0.05. Four connected nodes were identified. These genes also had significant betweenness and degree centrality. PTEN was the first tumor suppressor gene to be identified in the phosphatase family, and the principal function of its gene product appears to be dephosphorylation of the second messenger PIP3 [43]. The expression of PTEN in two independent glioblastoma cell lines results in the disruption of downstream signaling of PI3K to Akt and Bad [44]. Thus, when PTEN is present, Akt phosphorylation is blocked and apoptosis mechanisms may be activated. The importance of Akt and PTEN genes are as well revealed by this work, which illustrates the accuracy and efficiency of our algorithm.

As indicated in Figure 4, the connected gene DVL (disheveled, dsh homolog) connects two critical pathways: the WNT signaling pathway and the Notch signaling pathway. Gatcliffe *et al*. suggested that WNT signaling plays a role in ovarian tumorigenesis [45]. The WNT pathway participates in many physiologic events in embryogenesis and adult homeostasis including cell fate specification, control of proliferation, and migration. WNT signaling has a significant influence on the embryonic development of the ovary and is also involved in normal follicular development and ovarian function [46,47]. The WNT signaling pathway is involved in ovarian cancer development via multiple, diverse mechanisms, including gene mutations and changes in pathway components such as extracellular inhibitors and intranuclear transcription cofactors. According to Wang *et al*., the WNT signaling pathway passes signals to the Notch signaling pathway [48]. The Notch signaling pathway is known to be responsible for maintaining a balance between cell proliferation and death and, as such, plays an important role in the formation of many types of human tumors. In our computational results, WNT signaling connects the Notch signaling pathway through DVL gene, which indicates DVL is a critical gene for passing signals through pathways. In addition, the computational evidence provided by the values of betweenness centrality, degree and *p*-value indicate that DVL may be involved in platinum-based chemoresistance.

### The signature chemoresistance-associated genes

**Figure 5:**
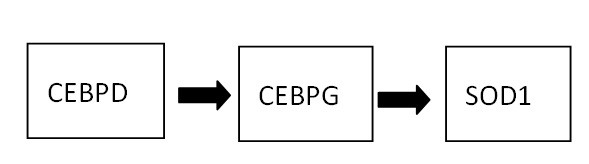
**The shortest pathway identified by our system**. Pathways including the gene CEBPD and SOD1 were the shortest pathways in our computational results, which indicates SOD1 (*p*-value = 4.01E-04) does not interact with other genes or pathways. We were curious about what caused the chemoresistant mechanism after SOD1 was regulated. Cisplatin caused DNA damage as well as reactive oxygen species (ROS), which triggered cell cycle arrest or/and apoptosis. Cisplatin induced CEBPD by an as of yet unidentified mechanism which activated the SOD1 gene expression. Superoxide anion (O2^•-^) is dismutated by SOD1 and converted to H_2_O_2 _which can be further neutralized to water and oxygen by catalase [23]. The reduced ROS levels in their model caused the cisplatin-resistant phenotype. These results call for an assessment of CEBPD and SOD1 expression in bladder tumors as a potential means of predicting cisplatin resistance. According to our computational results, SOD1 has significant differential expressions between chemosensitive and chemoresistant array data and is activated by CEBPD as well. We may make a reasonable assumption that this phenomenon occurs in ovarian chemoresistance. Based on this biological evidence and our computational experiment results, we can infer that SOD1 plays a critical role in ovarian chemoresistance.

Most of the results analyzed in the previous section are supported by known biological evidence, which indicates that this work is able to predict candidate chemoresistance-associated genes. We were particularly interested in CEPBD (CCAAT/enhancer binding protein delta) and its transcriptional regulated gene, SOD1 (Cu/Zn-superoxide dismutase). Several reports have implicated CEBPD as a suppressor gene [26-29]. According to Hour *et al*., the expression of the CEPBD was induced by cisplatin and specifically elevated in a cisplatin resistant subline and transactivated SOD1 gene expression in the human bladder urothelial carcinoma NTUB1 cell line [23]. This study revealed a novel role for CEBPD in conferring drug resistance. Therefore, we suspected CEBPD is involved in ovarian and lung chemoresistance as well. Moreover, as shown in Figure 5, pathways including the gene CEBPD and SOD1 were the shortest pathways in our computational results, which indicates SOD1 (*p*-value = 4.01E-04) does not interact with other genes or pathways. We were curious about what caused the chemoresistant mechanism after SOD1 was regulated. Cisplatin caused DNA damage as well as reactive oxygen species (ROS), which triggered cell cycle arrest or/and apoptosis. Cisplatin induced CEBPD by an as of yet unidentified mechanism which activated the SOD1 gene expression. Superoxide anion (O2^•-^) is dismutated by SOD1 and converted to H_2_O_2 _which can be further neutralized to water and oxygen by catalase [23]. The reduced ROS levels in their model caused the cisplatin-resistant phenotype. These results call for an assessment of CEBPD and SOD1 expression in bladder tumors as a potential means of predicting cisplatin resistance. According to our computational results, SOD1 has significant differential expressions between chemosensitive and chemoresistant array data and is activated by CEBPD as well. Do the reduced ROS levels caused by SOD1 in ovarian chemotherapy results in the resistant phenotype as well? We may make a reasonable assumption that this phenomenon occurs in ovarian chemoresistance. Based on this biological evidence and our computational experiment results, we can infer that SOD1 plays a critical role in ovarian chemoresistance.

As shown in Figure 4, CEBPD interacts with KRAS as well and led to a domino effect that may cause chemoresistance. It was found that mutations in this candidate gene, KRAS, are one of the most frequent genetic abnormalities in ovarian carcinoma [49]. In other words, KRAS mutation is a common event in ovarian cancer primarily in carcinomas characterized by lower grade, lower FIGO stage, and mucinous histotype. The KRAS mutational status is not a prognostic factor for patients treated with standard therapy. However, in line with experience from colorectal cancer and NSCLC, it may prove important for predicting the response to EGFR-targeted therapies [50]. Thus far, there is no biological evidence directly indicating KRAS gene is involved in platinum-based chemoresistance but, from the computational experiment results, we may infer that KRAS plays a critical role in chemoresistance. More computational results with high scores of intersected pathways are provided in Additional file 4, and analysis of these data may reveal new chemoresistant mechanisms.

## Conclusions

Although platinum-based chemotherapeutic agents are widely used for the treatment of endometrial, cervical and breast cancers, chemoresistance caused by molecular mechanisms still remains a major therapeutic problem. The platinum-based anti-tumor agent is a DNA-reactive reagent which causes cell cycle arrest at various phases in the cell cycle and induces apoptosis. Hence, the drug active pathway plays an important role in drug resistance in the cellular system. It is also a very important issue in the identification and validation of drug target genes by supplying their interactive relationships. This approach elucidated the particular chemoresistance-associated pathways in large biological interaction networks. Genes deemed relevant for chemotherapy resistance were also likewise determined. After identifying the chemoresistance-associated pathways, the scoring procedure filtered the significant pathways according to the genes' differential expressions. Consequently, this allowed for the identification of dissimilarities between the responses of chemosensitivity to the chemoresistance expression cancer data. In particular, we identified genes and pathway components such as the Hedgehog signalling pathway, the WNT signalling pathway, and the notch signalling pathway, that are relevant to chemoresistance for ovarian and lung cancer. The advantage of comparison analysis is in recognizing the divergent and convergent mechanisms of chemoresistance between cancers. Through systems biology methods, biologists can perform a comprehensive survey to upon which to base hypothetical assumptions.

The advantages of pathway intersections analysis include: revealing whether different cancers have same chemoresistant mechanisms, and determining whether some common genes involved in these chemoresistant mechanisms. As expected, we observed a great deal of correspondence between the response interactions of ovarian and lung cancer expression data by intersecting pathways. The analysis of platinum-based chemotherapeutic agents revealed insights into common responses among the chemoresistant mechanisms as well as the candidate genes such as Bcl-2, AHR and, most importantly, SOD1. The results also indicate that the WNT signaling pathway, the Notch signaling pathway and the FAK pathway are involved in ovarian and lung chemoresistance. Therefore, further analysis of our computational experiment results may reveal additional chemoresistance mechanisms, which indicates this approach can anticipate target identification and chemoresistance in the future development of cancer drugs.

Pathways with a dissimilar response to that of known modes of biological action can be easily identified early in the drug development process to avert repeated and costly clinical trails. This approach reveals chemoresistance-associated pathways *in scilicon *and enables easier comparisons with the generated graphs. Furthermore, by exploring signature genes involved in chemoresistance mechanisms, this approach sheds light on how these genes or pathways interact with each other, and provides analysis of the betweenness centrality and degree values of genes in pathways. In summary, this method is sufficiently flexible to accommodate various types of biological network information and experimental data, and offers not only insights into the mechanisms of chemoresistance but also provides information on potential candidate target genes for future drug-development efforts.

## Competing interests

The authors declare that they have no competing interests.

## Authors' contributions

SYC carried out the design of study, participated in the system implementation, wrote the program codes and drafted the manuscript. AMH participated in analysis the experimental results and helped to draft the manuscript. JHC participated in drafting the manuscript and WSC participated in writing the program codes. All authors read and approved the final manuscript.

## Pre-publication history

The pre-publication history for this paper can be accessed here:

http://www.biomedcentral.com/1755-8794/4/23/prepub

## Supplementary Material

Additional file 1 **Pathway lists**. The pathways used in this study are shown in additional file 1. Click here for file

Additional file 2 **Representation of the notations used by this work**. This additional file demonstrates the notations used in pathway representation. Click here for file

Additional file 3 **Significant results following pathway intersections**. The main analysis of this experiment focused on whether different cancers have same chemoresistant mechanisms and whether these chemoresistant mechanisms share some genes in common. We demonstrated the concept and the numeric results in this supplementary file. Click here for file

Additional file 4 **Pathway intersection results and analysis**. We demonstrated another pathway intersection result. In this pathway, the start node and end node are NF-_K_B and CENTG2, respectively. Several sub-pathways were involved in this experimental result, such as Apoptosis, Focal adhesion, and Jak-STAT signal pathway. More detailed analysis was shown in this file. Click here for file
