# Supplementary material for: An integrative approach to identifying cancer chemoresistance-associated pathways
Source: BMC Med Genomics. 2011 Mar 24;4:23. doi: 10.1186/1755-8794-4-23 (PMC3070611; doi:10.1186/1755-8794-4-23)
Supplement: Additional file 1 — Pathway lists. The pathways used in this study are shown in additional file 1. [file 1755-8794-4-23-S1.DOC]

An integrative approach to identifying cancer chemoresistance-associated pathways

Shih-Yi Chao^1^, Jung-Hsien Chiang ^2^, A-Mei Huang^3^ and Woan-Shan Chang^2^

^1^ Department of Computer Science and Information Engineering, Ching Yun University, No. 229, Jiansing Road, Jhongli City, Taoyuan County 320, Taiwan.

^2^Department of Computer Science and Information Engineering, National Cheng Kung University, No. 1, University Road, Tainan City 701, Taiwan.

^3^Department of Biochemistry, Kaoshiung Medical University, Shih-Chuan 1st Road, Kaohsiung, 807, Taiwan

**Additional file 1 --Pathway lists**

Platinum-based anti-cancer drugs, including cisplatin and carboplatin, have been used clinically for nearly thirty years as part of the treatment of many types of cancers, such as ovarian cancer, lung cancer, and colorectal cancer. The cytotoxic lesion of these agents is thought to be the platinum intrastrand crosslink that forms on DNA and activates a number of signal transduction pathways [1]. The cytotoxic lesion of these agents also causes DNA damage, DNA replication and DNA repair, which is one of the bases for selection of pathways. According to Siddik [2], cancer cells become resistant to anti-cancer drugs by several mechanisms. One way is to pump drugs out of cells by increasing the activity of efflux pumps, such as ATP-dependent transporters. As a result, pathways related to ATP-dependent transporters and transporters related to drug resistant were also selected [3]. Alternatively, resistance can occur as a result of reduced drug influx — a mechanism reported for agents that ‘piggyback’ on intracellular carriers or enter the cell by means of endocytosis [2]. Therefore, cellular processes related pathways were selected as well. Siddik also demonstrated that disruptions in apoptotic signalling pathways (e.g. tp53) allowed cells to become resistant to drug-induced cell death [2], which indicated that apoptotic signalling and signalling molecules and interaction related pathways were also selected by this approach. Moreover, treatment with cisplatin or carboplatin resulted in the activation of complex signaling cascades in the cell [1][4]. Transcription factors activated by these cascades served to vary the gene expression pattern after treatment with cisplatin or carboplatin [5-6], which was one of the bases for selection of pathways as well. Finally, we demonstrate all pathways used by this approach in the next section.

- **Metabolism**

1. **Carbohydrate Metabolism**
   - KEGG

hsa00010: [Glycolysis / Gluconeogenesis](http://www.genome.jp/kegg/pathway/map/map00010.html)

hsa00020: [Citrate cycle (TCA cycle)](http://www.genome.jp/kegg/pathway/map/map00020.html)

hsa00030: [Pentose phosphate pathway](http://www.genome.jp/kegg/pathway/map/map00030.html)

hsa00040: [Pentose and glucuronate interconversions](http://www.genome.jp/kegg/pathway/map/map00040.html)

hsa00051: [Fructose and mannose metabolism](http://www.genome.jp/kegg/pathway/map/map00051.html)

hsa00052: [Galactose metabolism](http://www.genome.jp/kegg/pathway/map/map00052.html)

hsa00053: [Ascorbate and aldarate metabolism](http://www.genome.jp/kegg/pathway/map/map00053.html)

hsa00500: [Starch and sucrose metabolism](http://www.genome.jp/kegg/pathway/map/map00500.html)

hsa00520: [Nucleotide sugars metabolism](http://www.genome.jp/kegg/pathway/map/map00520.html)

hsa00530: [Aminosugars metabolism](http://www.genome.jp/kegg/pathway/map/map00530.html)

hsa00562: [Inositol phosphate metabolism](http://www.genome.jp/kegg/pathway/map/map00562.html)

hsa00620: [Pyruvate metabolism](http://www.genome.jp/kegg/pathway/map/map00620.html)

hsa00630: [Glyoxylate and dicarboxylate metabolism](http://www.genome.jp/kegg/pathway/map/map00630.html)

hsa00650: [Butanoate metabolism](http://www.genome.jp/kegg/pathway/map/map00650.html)

hsa00640: [Propanoate metabolism](http://www.genome.jp/kegg/pathway/map/map00640.html)

1. **Lipid Metabolism**
   - KEGG

hsa00061: [Fatty acid biosynthesis](http://www.genome.jp/kegg/pathway/map/map00061.html)

hsa00062: [Fatty acid elongation in mitochondria](http://www.genome.jp/kegg/pathway/map/map00062.html)

hsa00071: [Fatty acid metabolism](http://www.genome.jp/kegg/pathway/map/map00071.html)

hsa00072: [Synthesis and degradation of ketone bodies](http://www.genome.jp/kegg/pathway/map/map00072.html)

hsa00100: [Biosynthesis of steroids](http://www.genome.jp/kegg/pathway/map/map00100.html)

hsa00120: [Bile acid biosynthesis](http://www.genome.jp/kegg/pathway/map/map00120.html)

hsa00140: [C21-Steroid hormone metabolism](http://www.genome.jp/kegg/pathway/map/map00140.html)

hsa00150: [Androgen and estrogen metabolism](http://www.genome.jp/kegg/pathway/map/map00150.html)

hsa00561: [Glycerolipid metabolism](http://www.genome.jp/kegg/pathway/map/map00561.html)

hsa00564: [Glycerophospholipid metabolism](http://www.genome.jp/kegg/pathway/map/map00564.html)

hsa00590: [Arachidonic acid metabolism](http://www.genome.jp/kegg/pathway/map/map00590.html)

hsa00600: [Sphingolipid metabolism](http://www.genome.jp/kegg/pathway/map/map00600.html)

hsa00601: [Glycosphingolipid biosynthesis - lacto and neolacto series](http://www.genome.jp/kegg/pathway/map/map00601.html)

hsa00602: Glycosphingolipid biosynthesis - neo-lactoseries

hsa00603: [Glycosphingolipid biosynthesis - globo series](http://www.genome.jp/kegg/pathway/map/map00603.html)

hsa00604: [Glycosphingolipid biosynthesis - ganglio series](http://www.genome.jp/kegg/pathway/map/map00604.html)

hsa00561: [Glycerolipid metabolism](http://www.genome.jp/kegg/pathway/map/map00561.html)

1. **Metabolism of Cofactors and Vitamins**
   - KEGG

hsa00130: [Ubiquinone and menaquinone biosynthesis](http://www.genome.jp/kegg/pathway/map/map00130.html)

hsa00670: [One carbon pool by folate](http://www.genome.jp/kegg/pathway/map/map00670.html)

hsa00730: [Thiamine metabolism](http://www.genome.jp/kegg/pathway/map/map00730.html)

hsa00740: [Riboflavin metabolism](http://www.genome.jp/kegg/pathway/map/map00740.html)

hsa00750: [Vitamin B6 metabolism](http://www.genome.jp/kegg/pathway/map/map00750.html)

hsa00760: [Nicotinate and nicotinamide metabolism](http://www.genome.jp/kegg/pathway/map/map00760.html)

hsa00770: [Pantothenate and CoA biosynthesis](http://www.genome.jp/kegg/pathway/map/map00770.html)

hsa00780: [Biotin metabolism](http://www.genome.jp/kegg/pathway/map/map00780.html)

hsa00790: [Folate biosynthesis](http://www.genome.jp/kegg/pathway/map/map00790.html)

hsa00860: [Porphyrin and chlorophyll metabolism](http://www.genome.jp/kegg/pathway/map/map00860.html)

1. **Amino Acid Metabolism**
   - KEGG

hsa00220: [Urea cycle and metabolism of amino groups](http://www.genome.jp/kegg/pathway/map/map00220.html)

hsa00252: [Alanine and aspartate metabolism](http://www.genome.jp/kegg/pathway/map/map00252.html)

hsa00260: [Glycine, serine and threonine metabolism](http://www.genome.jp/kegg/pathway/map/map00260.html)

hsa00271: [Methionine metabolism](http://www.genome.jp/kegg/pathway/map/map00271.html)

hsa00272: [Cysteine metabolism](http://www.genome.jp/kegg/pathway/map/map00272.html)

hsa00280: [Valine, leucine and isoleucine degradation](http://www.genome.jp/kegg/pathway/map/map00280.html)

hsa00290: [Valine, leucine and isoleucine biosynthesis](http://www.genome.jp/kegg/pathway/map/map00290.html)

hsa00300: [Lysine biosynthesis](http://www.genome.jp/kegg/pathway/map/map00300.html)

hsa00310: [Lysine degradation](http://www.genome.jp/kegg/pathway/map/map00310.html)

hsa00330: [Arginine and proline metabolism](http://www.genome.jp/kegg/pathway/map/map00330.html)

hsa00340: [Histidine metabolism](http://www.genome.jp/kegg/pathway/map/map00340.html)

hsa00350: [Tyrosine metabolism](http://www.genome.jp/kegg/pathway/map/map00350.html)

hsa00360: [Phenylalanine metabolism](http://www.genome.jp/kegg/pathway/map/map00360.html)

hsa00380: [Tryptophan metabolism](http://www.genome.jp/kegg/pathway/map/map00380.html)

1. **Nucleotide Metabolism**
   - KEGG

hsa00230: [Purine metabolism](http://www.genome.jp/kegg/pathway/map/map00230.html)

hsa00240: [Pyrimidine metabolism](http://www.genome.jp/kegg/pathway/map/map00240.html)

1. **Xenobiotics Biodegradation and Metabolism**
   - KEGG

hsa00361: [gamma-Hexachlorocyclohexane degradation](http://www.genome.jp/kegg/pathway/map/map00361.html)

hsa00627: [1,4-Dichlorobenzene degradation](http://www.genome.jp/kegg/pathway/map/map00627.html)

hsa00641: [3-Chloroacrylic acid degradation](http://www.genome.jp/kegg/pathway/map/map00641.html)

hsa00632: [Benzoate degradation via CoA ligation](http://www.genome.jp/kegg/pathway/map/map00632.html)

hsa00930: [Caprolactam degradation](http://www.genome.jp/kegg/pathway/map/map00930.html)

hsa00982: [Drug metabolism - cytochrome P450](http://www.genome.jp/kegg/pathway/map/map00982.html)

hsa00983: [Drug metabolism - other enzymes](http://www.genome.jp/kegg/pathway/map/map00983.html)

1. **Metabolism of Other Amino Acids**
   - KEGG

hsa00410: [beta-Alanine metabolism](http://www.genome.jp/kegg/pathway/map/map00410.html)

hsa00430: [Taurine and hypotaurine metabolism](http://www.genome.jp/kegg/pathway/map/map00430.html)

hsa00450: [Selenoamino acid metabolism](http://www.genome.jp/kegg/pathway/map/map00450.html)

hsa00460: [Cyanoamino acid metabolism](http://www.genome.jp/kegg/pathway/map/map00460.html)

hsa00471: [D-Glutamine and D-glutamate metabolism](http://www.genome.jp/kegg/pathway/map/map00471.html)

hsa00472: [D-Arginine and D-ornithine metabolism](http://www.genome.jp/kegg/pathway/map/map00472.html)

hsa00480: [Glutathione metabolism](http://www.genome.jp/kegg/pathway/map/map00480.html)

1. **Glycan Biosynthesis and Metabolism**
   - KEGG

hsa00510: [N-Glycan biosynthesis](http://www.genome.jp/kegg/pathway/map/map00510.html)

hsa00511: [Other glycan degradation](http://www.genome.jp/kegg/pathway/map/map00511.html)

hsa00512: [O-Glycan biosynthesis](http://www.genome.jp/kegg/pathway/map/map00512.html)

hsa00531: [Glycosaminoglycan degradation](http://www.genome.jp/kegg/pathway/map/map00531.html)

hsa00532: [Chondroitin sulfate biosynthesis](http://www.genome.jp/kegg/pathway/map/map00532.html)

hsa00533: [Keratan sulfate biosynthesis](http://www.genome.jp/kegg/pathway/map/map00533.html)

hsa00534: [Heparan sulfate biosynthesis](http://www.genome.jp/kegg/pathway/map/map00534.html)

hsa00550: [Peptidoglycan biosynthesis](http://www.genome.jp/kegg/pathway/map/map00550.html)

1. **Energy Metabolism**
   - KEGG

hsa00680: [Methane metabolism](http://www.genome.jp/kegg/pathway/map/map00680.html)

hsa00720: [Reductive carboxylate cycle (CO2 fixation)](http://www.genome.jp/kegg/pathway/map/map00720.html)

hsa00910: [Nitrogen metabolism](http://www.genome.jp/kegg/pathway/map/map00910.html)

hsa00920: [Sulfur metabolism](http://www.genome.jp/kegg/pathway/map/map00920.html)

1. **Biosynthesis of Secondary Metabolites**
   - KEGG

hsa00900: [Terpenoid biosynthesis](http://www.genome.jp/kegg/pathway/map/map00900.html)

hsa00902: [Monoterpenoid biosynthesis](http://www.genome.jp/kegg/pathway/map/map00902.html)

hsa00903: [Limonene and pinene degradation](http://www.genome.jp/kegg/pathway/map/map00903.html)

hsa00950: [Alkaloid biosynthesis I](http://www.genome.jp/kegg/pathway/map/map00950.html)

hsa00960: [Alkaloid biosynthesis II](http://www.genome.jp/kegg/pathway/map/map00960.html)

#### Genetic Information Processing

1. **Transcription**
   - KEGG

hsa03020: [RNA polymerase](http://www.genome.jp/kegg/pathway/ko/ko03020.html)

1. **Replication and Repair**
   - KEGG

hsa03030: [DNA replication](http://www.genome.jp/kegg/pathway/ko/ko03030.html)

hsa03410: [Base excision repair](http://www.genome.jp/kegg/pathway/ko/ko03410.html)

hsa03420: [Nucleotide excision repair](http://www.genome.jp/kegg/pathway/ko/ko03420.html)

hsa03430: [Mismatch repair](http://www.genome.jp/kegg/pathway/ko/ko03430.html)

hsa03440: [Homologous recombination](http://www.genome.jp/kegg/pathway/ko/ko03440.html)

- - PID

DNA-replication initiation

ATM mediated response to DNA double-strand break assemble of the RAD50-MRE11-NBS1 complex at DNA double-strand breaks

DNA damage bypass

DNA damage recognition on GG-NER

DNA damage Reversal1

Gap-filling DNA repair synthesis and ligation in GG-NER

Gap-filling DNA repair synthesis and ligation in TC-NER

Homologous DNA pairing and strand exchange

Homologous recombination reapir

homologous recombination repair of replication-independent double-strand breaks

MRN complex relocalizes to unclear foci

non homologous end-joining (NHEJ)

nucleotide excision reapir

presynaptic phase of homologous DNA pairing and strand exchange

processing of DNA double-strand break ends

processing of DNA ends prior to end rejoining

Recognition and association of DNA glycosylase with site containing an affected purine

Recruitment of repair and signaling proteins to double-strand breaks

Removal of DNA patch containing abasic residue

Repair synthesis for gap-filling by DNA polymerase in TC-NER

Repair synthesis of patch ~27-30 bases long by DNA polymerase

Resolution of AP sites via the multiple-nucleotide patch replacement pathway

Resolution_of_AP_sites_via_the_single_nucleotide_replacement_pathway

Transcription_coupled_NER__TC_NER_

Translesion_synthesis_by_DNA_polymerases_bypassing_lesion_on_DNA_template

Activation_of_the_pre_replicative_complex

Assembly_of_the_ORC_complex_at_the_origin_of_replication

Assembly_of_the_pre_replicative_complex

Association_of_licensing_factors_with_the_pre_replicative_complex

CDC6_association_with_the_ORC_origin_complex

CDK-mediated phosphorylation and removal of CDC6

CDT1_association_with_the_CDC6_ORC_origin_complex

DNA_Replication_Pre_Initiation

DNA_replication_initiation

DNA_strand_elongation

Lagging_Strand_Synthesis

Leading_Strand_Synthesis

Orc1_removal_from_chromatin

Polymerase_switching

Processive_synthesis_on_the_lagging_strand

Regulation_of_DNA_replication

Removal_of_licensing_factors_from_origins

Removal_of_the_Flap_Intermediate

Switching_of_origins_to_a_post_replicative_state

Unwinding_of_DNA

1. **Folding, Sorting and Degradation**
   - KEGG

hsa03050: [Proteasome](http://www.genome.jp/kegg/pathway/ko/ko03050.html)

hsa04120: [Ubiquitin mediated proteolysis](http://www.genome.jp/kegg/pathway/ko/ko04120.html)

hsa04140: [Regulation of autophagy](http://www.genome.jp/kegg/pathway/ko/ko04140.html)

1. **Signal Transduction**
   - KEGG

hsa04010: [MAPK signaling pathway](http://www.genome.jp/kegg/pathway/hsa/hsa04010.html)

hsa04012: [ErbB signaling pathway](http://www.genome.jp/kegg/pathway/hsa/hsa04012.html)
hsa04310: [Wnt signaling pathway](http://www.genome.jp/kegg/pathway/hsa/hsa04310.html)
hsa04330: [Notch signaling pathway](http://www.genome.jp/kegg/pathway/hsa/hsa04330.html)
hsa04340: [Hedgehog signaling pathway](http://www.genome.jp/kegg/pathway/dme/dme04340.html)
hsa04350: [TGF-beta signaling pathway](http://www.genome.jp/kegg/pathway/hsa/hsa04350.html)
hsa04370: [VEGF signaling pathway](http://www.genome.jp/kegg/pathway/hsa/hsa04370.html)
hsa04630: [Jak-STAT signaling pathway](http://www.genome.jp/kegg/pathway/hsa/hsa04630.html)
hsa04020: [Calcium signaling pathway](http://www.genome.jp/kegg/pathway/hsa/hsa04020.html)
hsa04070: [Phosphatidylinositol signaling system](http://www.genome.jp/kegg/pathway/hsa/hsa04070.html)
hsa04150: [mTOR signaling pathway](http://www.genome.jp/kegg/pathway/hsa/hsa04150.html)

#### Cellular Processes

1. **Cell Growth and Death**
   - KEGG

hsa04110: [Cell cycle](http://www.genome.jp/kegg/pathway/hsa/hsa04110.html)

hsa04210: [Apoptosis](http://www.genome.jp/kegg/pathway/hsa/hsa04210.html)

hsa04115: [p53 signaling pathway](http://www.genome.jp/kegg/pathway/hsa/hsa04115.html)

- - PID

Apoptotic signaling in response to DNA damage

cadmium induces DNA synthesis and proliferation in macrophages

apoptic DNA-fragementation and tissue homeostasis

cdc25 and chk1 regulatory pathway in response to DNA damage

cdk regulation of DNA replication

cell cycle G1/S check point

cell cycle G2/M checkpoint

Rb turmor suppressor/checkpoint signaling in response to DNA damage

regulation of cell cycle progression by plk3

G1/S dna DAMAGE CHECKPOINTS

G2/M DNA damage checkpoint

G2/M DNA replication checkpoint

p53-dependent G1 DAN damage

p53-dependent G1/S DNA damage

p53-independent DNA damage response

p53-independent G1/S DNA damage

1. **Cell Motility**
   - KEGG

hsa04810: [Regulation of actin cytoskeleton](http://www.genome.jp/kegg/pathway/hsa/hsa04810.html)

1. **Cell Communication**
   - KEGG

hsa04510: [Focal adhesion](http://www.genome.jp/kegg/pathway/hsa/hsa04510.html)
hsa04520: [Adherens junction](http://www.genome.jp/kegg/pathway/hsa/hsa04520.html)
hsa04530: [Tight junction](http://www.genome.jp/kegg/pathway/hsa/hsa04530.html)

1. **Immune System**
   - KEGG

hsa04610: [Complement and coagulation cascades](http://www.genome.jp/kegg/pathway/hsa/hsa04610.html)

hsa04662: [B cell receptor signaling pathway](http://www.genome.jp/kegg/pathway/hsa/hsa04662.html)

1. **Nervous System**
   - KEGG

hsa04720: [Long-term potentiation](http://www.genome.jp/kegg/pathway/hsa/hsa04720.html)
hsa04730: [Long-term depression](http://www.genome.jp/kegg/pathway/hsa/hsa04730.html)

1. **Endocrine System**
   - KEGG

hsa04910: [Insulin signaling pathway](http://www.genome.jp/kegg/pathway/hsa/hsa04910.html)

#### Human Diseases

1. **Cancers**
   - KEGG

hsa05210: [Colorectal cancer](http://www.genome.jp/kegg/pathway/hsa/hsa05210.html)
hsa05212: [Pancreatic cancer](http://www.genome.jp/kegg/pathway/hsa/hsa05212.html)
hsa05214: [Glioma](http://www.genome.jp/kegg/pathway/hsa/hsa05214.html)
hsa05216: [Thyroid cancer](http://www.genome.jp/kegg/pathway/hsa/hsa05216.html)
hsa05221: [Acute myeloid leukemia](http://www.genome.jp/kegg/pathway/hsa/hsa05221.html)
hsa05220: [Chronic myeloid leukemia](http://www.genome.jp/kegg/pathway/hsa/hsa05220.html)
hsa05217: [Basal cell carcinoma](http://www.genome.jp/kegg/pathway/hsa/hsa05217.html)
hsa05218: [Melanoma](http://www.genome.jp/kegg/pathway/hsa/hsa05218.html)
hsa05211: [Renal cell carcinoma](http://www.genome.jp/kegg/pathway/hsa/hsa05211.html)
hsa05219: [Bladder cancer](http://www.genome.jp/kegg/pathway/hsa/hsa05219.html)
hsa05215: [Prostate cancer](http://www.genome.jp/kegg/pathway/hsa/hsa05215.html)
hsa05213: [Endometrial cancer](http://www.genome.jp/kegg/pathway/hsa/hsa05213.html)
hsa05222: [Small cell lung cancer](http://www.genome.jp/kegg/pathway/hsa/hsa05222.html)
hsa05223: [Non-small cell lung cancer](http://www.genome.jp/kegg/pathway/hsa/hsa05223.html)

#### Environmental Information Processing

1. **Signaling Molecules and Interaction**
   - KEGG

hsa04060: [Cytokine-cytokine receptor interaction](http://www.genome.jp/kegg/pathway/hsa/hsa04060.html)

hsa04512: [ECM-receptor interaction](http://www.genome.jp/kegg/pathway/hsa/hsa04512.html)

Reference

[1]Rabik CA, Dolan M: **Molecular mechanisms of resistance and toxicity associated with platinating agents**. *Cancer treatment reviews* 2007, **33**: 9-23.

[2] Siddik ZH: **Cisplatin: mode of cytotoxic action and molecular basis of resistance**. *Oncogene* 2003, **22:**7265-7279.

[3] Hembruff SL, Laberge ML, Villeneuve DJ, Guo B, Veitch Z, Cecchetto M, Parissenti AM: **Role of drug transporters and drug accumulation in the temporal acquisition of drug resistance**. *BMC* *Cancer* 2008, **8:**318–334.

[4] Kartalou M, Essigmann JM: **Mechanisms of resistance to cisplatin**. *Mutation Research* 2001, **478:**23–43.

[5] Torigoe T, Izumi H, Ishiguchi H, Yoshida Y, Tanabe M, Yoshida T: **Cisplatin resistance and transcription factors**. *Current Medicinal Chemistry. Anti-Cancer Agents* 2005, **5:**15-27.

[6] Basu A and Krishnamurthy S: **Cellular Responses to Cisplatin-Induced DNA** **Damage**. *Journal of Nucleic Acids* 2010, article ID 201367, 16 pages, doi:10.4061/2010/201367.
