# Supplementary material for: An integrative approach to identifying cancer chemoresistance-associated pathways
Source: BMC Med Genomics. 2011 Mar 24;4:23. doi: 10.1186/1755-8794-4-23 (PMC3070611; doi:10.1186/1755-8794-4-23)
Supplement: Additional file 2 — Representation of the notations used by this work. This additional file demonstrates the notations used in pathway representation. [file 1755-8794-4-23-S2.DOC]

An integrative approach to identifying cancer chemoresistance-associated pathways

Shih-Yi Chao^1^, Jung-Hsien Chiang ^2^, A-Mei Huang^3^ and Woan-Shan Chang^2^

^1^ Department of Computer Science and Information Engineering, Ching Yun University, No. 229, Jiansing Road, Jhongli City, Taoyuan County 320, Taiwan.

^2^Department of Computer Science and Information Engineering, National Cheng Kung University, No. 1, University Road, Tainan City 701, Taiwan.

^3^Department of Biochemistry, Kaoshiung Medical University, Shih-Chuan 1st Road, Kaohsiung, 807, Taiwan

**Additional file 2 –Representation of the notations used by this work**

The squares represent gene products and the grey arrows indicate the activate relations parsed from TRANSFAC while black arrows from KEGG database.

Table 3. The notations used in pathway representation.

| **Objects** | | **description** |
| --- | --- | --- |
|  | Gene product, mostly protein but including RNA | |
|  | Other molecule, mostly chemical compound | |
|  | Another pathway | |
| **Gene expression relations** | | **description** |
|  | expression | |
|  | repression | |
| **e** | expression | |

| **Arrows** | **description** |
| --- | --- |
|  | Activation (relations from KEGG database) |
| **+p** | Activation (relations from TRANSFAC database) |
|  | Phosphorylation |
| **-p**  **+u** | Dephosphorylation |
|  | Ubiquitination |
| **+g** | glycosylation |
| **+m** | methylation |
