# Supplementary material for: An integrative approach to identifying cancer chemoresistance-associated pathways
Source: BMC Med Genomics. 2011 Mar 24;4:23. doi: 10.1186/1755-8794-4-23 (PMC3070611; doi:10.1186/1755-8794-4-23)
Supplement: Additional file 3 — Significant results following pathway intersections. The main analysis of this experiment focused on whether different cancers have same chemoresistant mechanisms and whether these chemoresistant mechanisms share some genes in common. We demonstrated the concept and the numeric results in this supplementary file. [file 1755-8794-4-23-S3.DOC]

An integrative approach to identifying cancer chemoresistance-associated pathways

Shih-Yi Chao^1^, Jung-Hsien Chiang ^2^, A-Mei Huang^3^ and Woan-Shan Chang^2^

^1^ Department of Computer Science and Information Engineering, Ching Yun University, No. 229, Jiansing Road, Jhongli City, Taoyuan County 320, Taiwan.

^2^Department of Computer Science and Information Engineering, National Cheng Kung University, No. 1, University Road, Tainan City 701, Taiwan.

^3^Department of Biochemistry, Kaoshiung Medical University, Shih-Chuan 1st Road, Kaohsiung, 807, Taiwan

**Additional file 3 - Significant results following pathway intersections**

The main analysis of this experiment focused on whether different cancers have same chemoresistant mechanisms and whether these chemoresistant mechanisms share some genes in common. To achieve this goal, we chose pathways with top n (=3) highest score of each pairing seed nodes resulting from ovarian cancer (430 pathways) and lung cancer (188 pathways) respectively. After performing intersection, 88 pathways remained.


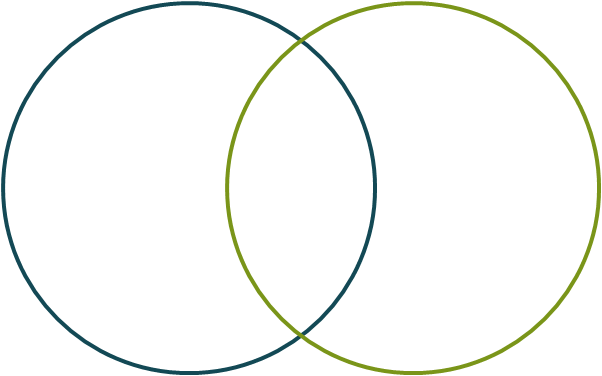


After ranking top n (=3) highest score, 430 pathways were identified from ovarian cancer expression data

After ranking top n (=3) highest score, 188 pathways were identified from NSCLC cancer expression data

**Intersection: 88**

Figure 1. The results of performing intersection pathways

We provide general tables with summary of pathway members and scores (the highest score from ovarian and lung cancer data, respectively).

- Start node：ATF4，end node：CYP27C1

| **Same shortest paths** | **Ovarian Score** | **NSCLC Score** |
| --- | --- | --- |
| CREB3,CREB1,CREB3L4,ATF4,CREB3L2,CREB3L3,CREB3L1,CREB5 → CREBBP, RSTS,EP300 → TP53~PAX5 → PTEN, BZS, MHAM → AKT3,AKT1,AKT2 → MDM2 → TP53~HOXA9 → FAS → DAXX → MAP3K5 → MAP2K6 → MAPK14, CSPB1, CSBP1, CSBP2,MAPK11, PRKM11,MAPK13, PRKM13,MAPK12, SAPK3 → DDIT3 → CYP27C1 | 1.65 | 3.61 |

- Start node：ATF4，end node：RBMS3

| **Same shortest paths** | **Ovarian Score** | **NSCLC Score** |
| --- | --- | --- |
| CREB3,CREB1,CREB3L4,ATF4,CREB3L2,CREB3L3,CREB3L1,CREB5 → CREBBP, RSTS,EP300 → TP53~PAX5 → PTEN, BZS, MHAM → AKT3,AKT1,AKT2 → MDM2 → TP53 → RBMS3 | 1.629 | 2.25 |

- Start node：ATF4，end node：PCNA

| **Same shortest paths** | **Ovarian Score** | **NSCLC Score** |
| --- | --- | --- |
| CREB3,CREB1,CREB3L4,ATF4,CREB3L2,CREB3L3,CREB3L1,CREB5 → CREBBP, RSTS,EP300 → TP53~PAX5 → MDM2 → TP53~HOXA9 → PTEN, BZS, MHAM → AKT3,AKT1,AKT2 → MAP2K4, SERK1 → MAPK8, PRKM8,MAPK9, PRKM9,MAPK10, PRKM10 → TP53~TFAP4 → GADD45G,GADD45A, DDIT1,GADD45B → PCNA | 3.67 | 2.34 |

- Start node：ATF4，end node：SCAPER

| **Same shortest paths** | **Ovarian Score** | **NSCLC Score** |
| --- | --- | --- |
| CREB3,CREB1,CREB3L4,ATF4,CREB3L2,CREB3L3,CREB3L1,CREB5 → CREBBP, RSTS,EP300 → TP53~PAX5 → PTEN, BZS, MHAM → AKT3,AKT1,AKT2 → MDM2 → TP53~HOXA9 → FAS → DAXX → MAP3K5 → MAP2K6 → MAPK14, CSPB1, CSBP1, CSBP2,MAPK11, PRKM11,MAPK13, PRKM13,MAPK12, SAPK3 → ATF2 → SCAPER | 1.649 | 3.604 |

- Start node：ATF4，end node：TP73

| **Same shortest paths** | **Ovarian Score** | **NSCLC Score** |
| --- | --- | --- |
| CREB3,CREB1,CREB3L4,ATF4,CREB3L2,CREB3L3,CREB3L1,CREB5 → CREBBP, RSTS,EP300 → TP53~PAX5 → MDM2 → TP53~HOXA9 → PTEN, BZS, MHAM → AKT3,AKT1,AKT2 → MAP2K4, SERK1 → MAPK8, PRKM8,MAPK9, PRKM9,MAPK10, PRKM10 → TP53~TFAP4 → TP73 | 2.79 | 1.70 |

- Start node：CEBPD，end node：PCNA

| **Same shortest paths** | **Ovarian Score** | **NSCLC Score** |
| --- | --- | --- |
| CEBPD → CHEK1 → TP53~HOXA9 → GADD45G,GADD45A, DDIT1,GADD45B → PCNA | 2.039 | 2.02 |

- Start node：CEBPD，end node：TP73

| **Same shortest paths** | **Ovarian Score** | **NSCLC Score** |
| --- | --- | --- |
| CEBPD → CHEK1 → TP53~HOXA9 → TP73 | 0.98 | 2.046 |

- Start node：CEBPD，end node：ZMIZ1

| **Same shortest paths** | **Ovarian Score** | **NSCLC Score** |
| --- | --- | --- |
| CEBPD → CHD8, HELSNF1 → CTNNB1, CTNNB → CREBBP, RSTS,EP300 → TP53~HOXA9 → PTEN, BZS, MHAM → PTK2 → SHC2,SHC4,SHC3,SHC1, SHC → GRB2 → SOS1, GINGF,SOS2 → MYC → ZMIZ1 | 2.86 | 0.211 |

- Start node：CUTL1，end node：CENTG2

| **Same shortest paths** | **Ovarian Score** | **NSCLC Score** |
| --- | --- | --- |
| CUTL1 → JUP, CTNNG → MYC → CENTG2 | 2.299 | 0.933 |

- Start node：CUTL1，end node：NFKB2

| **Same shortest paths** | **Ovarian Score** | **NSCLC Score** |
| --- | --- | --- |
| CUTL1 → NFKBIA → NFKB1,NFKB2,RELA | 4.193 | 0.866 |

- Start node：CUTL1，end node：PCNA

| **Same shortest paths** | **Ovarian Score** | **NSCLC Score** |
| --- | --- | --- |
| CUTL1 → MAP2K1, PRKMK1 → MAPK8, PRKM8,MAPK9, PRKM9,MAPK10, PRKM10 → TP53~PAX5 → GADD45G,GADD45A, DDIT1,GADD45B → PCNA | 2.5197 | 0.129 |

- Start node：IL1B，end node：CYP27C1

| **Same shortest paths** | **Ovarian Score** | **NSCLC Score** |
| --- | --- | --- |
| IL1A,IL1B → IL1R1,IL1R2 → CASP3 → MAP3K1, MEKK1 → MAP2K1, PRKMK1 → MAPK8, PRKM8,MAPK9, PRKM9,MAPK10, PRKM10 → TP53~HOXA9 → FAS → DAXX → MAP3K5 → MAP2K3 → MAPK14, CSPB1, CSBP1, CSBP2,MAPK11, PRKM11,MAPK13, PRKM13,MAPK12, SAPK3 → DDIT3 → CYP27C1 | 2.059 | 0.721 |

- Start node：IL1B，end node：RBMS3

| **Same shortest paths** | **Ovarian Score** | **NSCLC Score** |
| --- | --- | --- |
| IL1A,IL1B → IL1R1,IL1R2 → CASP3 → MAP3K1, MEKK1 → MAP2K1, PRKMK1 → MAPK1, PRKM2, PRKM1,MAPK3, PRKM3 → BCL2 → TP53~PAX5 → MDM2 → TP53 → RBMS3 | 1.547 | 1.386 |

- Start node：IL1A，end node：TP73

| **Same shortest paths** | **Ovarian Score** | **NSCLC Score** |
| --- | --- | --- |
| IL1A,IL1B → IL1R1,IL1R2 → CASP3 → MAP3K1, MEKK1 → MAP2K4, SERK1 → MAPK8, PRKM8,MAPK9, PRKM9,MAPK10, PRKM10 → TP53 → MDM2 → TP53~PAX5 → TP73 | 1.587 | 0.545 |

- Start node：IL1A，end node：SCAPER

| **Same shortest paths** | **Ovarian Score** | **NSCLC Score** |
| --- | --- | --- |
| IL1A,IL1B → IL1R1,IL1R2 → CASP3 → MAP3K1, MEKK1 → MAP2K1, PRKMK1 → MAPK8, PRKM8,MAPK9, PRKM9,MAPK10, PRKM10 → TP53~HOXA9 → FAS → DAXX → MAP3K5 → MAP2K3 → MAPK14, CSPB1, CSBP1, CSBP2,MAPK11, PRKM11,MAPK13, PRKM13,MAPK12, SAPK3 → ATF2 → SCAPER | 2.055 | 0.703 |

- Start node：NFKB1，end node：CENTG2

| **Same shortest paths** | **Ovarian Score** | **NSCLC Score** |
| --- | --- | --- |
| NFKB1,NFKB2,RELA → BCL2 → TP53~HOXA9 → PTEN, BZS, MHAM → PTK2 → SHC2,SHC4,SHC3,SHC1, SHC → GRB2 → SOS1, GINGF,SOS2 → MYC → CENTG2 | 3.23 | 0.904 |

- Start node：NFKB1，end node：JAZF1

| **Same shortest paths** | **Ovarian Score** | **NSCLC Score** |
| --- | --- | --- |
| NFKB1,NFKB2,RELA → BCL2 → TP53~HOXA9 → PTEN, BZS, MHAM → PTK2 → SHC2,SHC4,SHC3,SHC1, SHC → GRB2 → SOS1, GINGF,SOS2 → MYC → JAZF1 | 3.231 | 0.904 |

- Start node：NFKB1，end node：RBMS3

| **Same shortest paths** | **Ovarian Score** | **NSCLC Score** |
| --- | --- | --- |
| NFKB1,NFKB2,RELA → BCL2 → TP53~PAX5 → MDM2 → TP53 → RBMS3 | 0.742 | 3.648 |

- Start node：NFKB1，end node：PCNA

| **Same shortest paths** | **Ovarian Score** | **NSCLC Score** |
| --- | --- | --- |
| NFKB1,NFKB2,RELA → BCL2 → TP53~HOXA9 → GADD45G,GADD45A, DDIT1,GADD45B → PCNA | 1.872 | 2.13 |

- Start node：NFKB1，end node：CYP27C1

| **Same shortest paths** | **Ovarian Score** | **NSCLC Score** |
| --- | --- | --- |
| NFKB1,NFKB2,RELA → BIRC2, API1,BIRC3, API2,XIAP, API3, BIRC4 → CASP3 → MAP3K1, MEKK1 → MAP2K1, PRKMK1 → MAPK8, PRKM8,MAPK9, PRKM9,MAPK10, PRKM10 → TP53~PAX5 → FAS → DAXX → MAP3K5 → MAP2K3 → MAPK14, CSPB1, CSBP1, CSBP2,MAPK11, PRKM11,MAPK13, PRKM13,MAPK12, SAPK3 → DDIT3 → CYP27C1 | 1.704 | 0.262 |

- Start node：NFKB2，end node：SCAPER

| **Same shortest paths** | **Ovarian Score** | **NSCLC Score** |
| --- | --- | --- |
| NFKB1,NFKB2,RELA → BIRC2, API1,BIRC3, API2,XIAP, API3, BIRC4 → CASP3 → MAP3K1, MEKK1 → MAP2K1, PRKMK1 → MAPK8, PRKM8,MAPK9, PRKM9,MAPK10, PRKM10 → TP53~PAX5 → FAS → DAXX → MAP3K5 → MAP2K3 → MAPK14, CSPB1, CSBP1, CSBP2,MAPK11, PRKM11,MAPK13, PRKM13,MAPK12, SAPK3 → ATF2 → SCAPER | 1.699 | 0.244 |

- Start node：NFKB2，end node：TP73

| **Same shortest paths** | **Ovarian Score** | **NSCLC Score** |
| --- | --- | --- |
| NFKB1,NFKB2,RELA → BCL2 → TP53~HOXA9 → TP73 | 0.8 | 1.491 |

- Start node：NFKB2，end node：ZMIZ1

| **Same shortest paths** | **Ovarian Score** | **NSCLC Score** |
| --- | --- | --- |
| NFKB1,NFKB2,RELA → BCL2 → TP53~HOXA9 → PTEN, BZS, MHAM → PTK2 → SHC2,SHC4,SHC3,SHC1, SHC → GRB2 → SOS1, GINGF,SOS2 → MYC → ZMIZ1 | 3.23 | 0.904 |
