# Supplementary material for: An integrative approach to identifying cancer chemoresistance-associated pathways
Source: BMC Med Genomics. 2011 Mar 24;4:23. doi: 10.1186/1755-8794-4-23 (PMC3070611; doi:10.1186/1755-8794-4-23)
Supplement: Additional file 4 — Pathway intersection results and analysis. We demonstrated another pathway intersection result. In this pathway, the start node and end node are NF-KB and CENTG2, respectively. Several sub-pathways were involved in this experimental result, such as Apoptosis, Focal adhesion, and Jak-STAT signal pathway. More detailed analysis was shown in this file. [file 1755-8794-4-23-S4.DOC]

An integrative approach to identifying cancer chemoresistance-associated pathways

Shih-Yi Chao^1^, Jung-Hsien Chiang ^2^, A-Mei Huang^3^ and Woan-Shan Chang^2^

^1^ Department of Computer Science and Information Engineering, Ching Yun University, No. 229, Jiansing Road, Jhongli City, Taoyuan County 320, Taiwan.

^2^Department of Computer Science and Information Engineering, National Cheng Kung University, No. 1, University Road, Tainan City 701, Taiwan.

^3^Department of Biochemistry, Kaoshiung Medical University, Shih-Chuan 1st Road, Kaohsiung, 807, Taiwan

**Additional file 4 – pathway intersection results and analysis**

We demonstrated another experimental result of pathway intersection. In this pathway, the start node and end node were NF-_K_B (nuclear factor of kappa light polypeptide gene enhancer in B-cells) and CENTG2 (centaurin gamma-2), respectively. Several pathways were involved in this experimental result, such as Apoptosis, Focal adhesion, and Jak-STAT signalling pathway. The protein tyrosine kinase focal adhesion kinase (FAK) played an important role in integrin signalling [1]. Activation of FAK resulted in recruitment of a number of SH2-domain- and SH3-domain-containing proteins, which mediated signals to several downstream pathways. FAK-dependent activation of these pathways had been implicated in a diverse array of cellular processes including cell migration, growth factor signalling, cell cycle progression and cell survival. Moreover, Fraser *et al.* suggested that in ovarian chemoresistant, FAK was activated and inhibited the mechanism of cell apoptosis [1]. The importance of Jak-Stat pathway signaling in regulating cytokine-dependent gene expression and cellular development/survival was well established. In addition, several studies had indicated that the Jak-Stat pathway signalling was related to several tumorigenesis [2]. Hong *et al*. indicated that BCL2 was important in cisplatin resistance and the decrease in BCL2 genes with antisense oligonucleotide can reverse cisplatin sensitivity [3]. Antisense BCL2 oligonucleotide may be a novel therapeutic strategy in the treatment of cisplatin-resistant. In accordance with our computational experiment results shown in Table 1, BCL2 as a significantly differential expression in ovarian and lung cancer not only played a critical role in regulating several genes but also supported the conclusion of BCL2, a candidate gene.


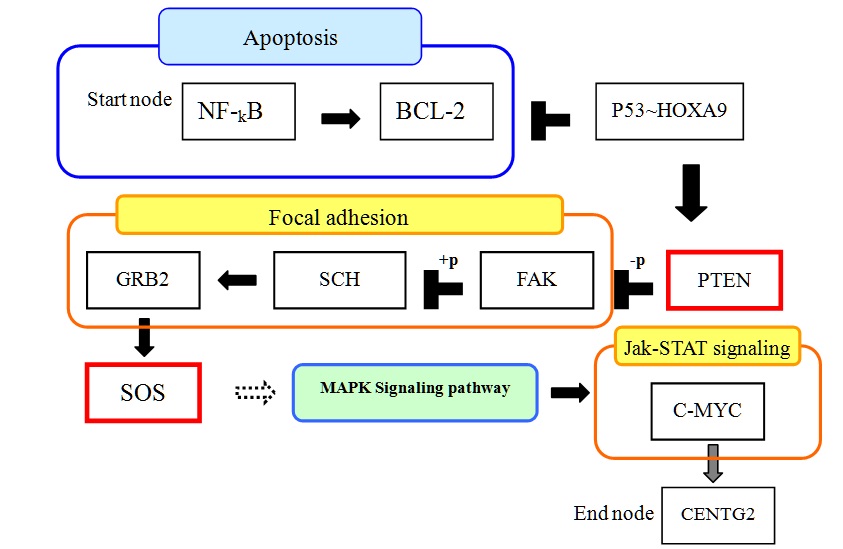


Figure 1. One of the experimental results from pathway intersection.

Table 1 Genes identified in figure 1 with *p*-value < 0.05 by t-test

| **Gene Symbol** | **ovarian**  ***p*-value** | **lung**  ***p*-value** | **Betweenness**  **(mean=3.8E-4)** | **Degree**  **(mean=9.71E-4)** | **Connected node** |
| --- | --- | --- | --- | --- | --- |
| BCL2  (B-cell CLL/lymphoma 2) | 4.41E-07 | 6.06E-05 | 0.006596 | 0.008228 |  |
| PTK2  (PTK2 protein tyrosine kinase 2) | 1.76E-04 | 2.81E-04 | 0.006291 | 0.009849 |  |
| SHC1  (SHC (Src homology 2 domain containing) transforming protein 1) | 9.85E-04 | N/A | 0.007758 | 0.014088 |  |
| GRB2  (growth factor receptor-bound protein 2) | 5.74E-06 | 1.01E-04 | 0.020155 | 0.023064 |  |
| SOS1  ( son of sevenless homolog 1 (Drosophila)) | 6.41E-05 | N/A | 0.001784 | 0.005486 | V |
| SOS2  ( son of sevenless homolog 2 (Drosophila)) | 3.46E-05 | 8.12E-04 | 1.27E-04 | 0.001621 | V |
| c-Myc  (v-myc myelocytomatosis viral oncogene homolog (avian)) | 0.001269 | 1.13E-06 | 0.004401 | 0.007979 |  |
| TP53  (tumor protein p53) | 0.011083 | 1.82E-04 | 0.046039 | 0.029049 | V |
| CENTG2  (AGAP1, ArfGAP with GTPase domain, ankyrin repeat and PH domain 1) | 0.005123 | N/A | 2.43E-04 | 2.49E-04 |  |

**Reference**

1. Fraser M, Leung B, Jahani-Asl A, Yan X, Thompson WE, Tsang BK: **Chemoresistance in human ovarian cancer: the role of apoptotic regulators***.* *Reproductive Biology and Endocrinology* 2003, **1**: 66-79.
2. O'Shea JJ, Gadina M, Schreiber RD: **Cytokine Signaling in 2002: New Surprises in the Jak/Stat Pathway**. *Cell* 2002, **109**: S121-S131.
3. Hong JH, Lee E, Hong J, Shin YJ, Ahn H: **Antisense Bcl2 oligonucleotide in cisplatin-resistant bladder cancer cell lines**. *British Journal of Urology International* 2002, **90**: 113-117.
